# Impact of Transitory ROSC Events on Neurological Outcome in Patients with Out-of-Hospital Cardiac Arrest

**DOI:** 10.3390/jcm8070926

**Published:** 2019-06-27

**Authors:** Vittorio Antonaglia, Carlo Pegani, Giuseppe Davide Caggegi, Athina Patsoura, Veronica Xu, Marco Zambon, Gianfranco Sanson

**Affiliations:** 1 Regional Emergency Medical Service System, Azienda Regionale Coordinamento della Salute, 33057 Udine, Italy; 2 Emergency Medical Service System, Azienda Sanitaria Universitaria Integrata, 734128 Trieste, Italy; 3 Department of Surgery, Dentistry, Paediatrics and Gynaecology, School of Medicine, University of Verona, 837129 Verona, Italy; 4 Azienda USL Toscana Sud Est, 54-52100 Arezzo, Italy; 5 Department of Medicine, Surgery and Health Sciences, University of Trieste, 9-34100 Piazzale Europa, Italy

**Keywords:** cardiac arrest, cardio-pulmonary resuscitation, cerebral performance category, temporary ROSC, out-of-hospital, quality metrics, outcome

## Abstract

In out-of-hospital cardiac arrest (OHCA), the occurrence of temporary periods of return to spontaneous circulation (t-ROSC) has been found to be predictive of survival to hospital discharge. The relationship between the duration of t-ROSCs and OHCA outcome has not been explored yet. The aim of this prospective observational study was to analyze the duration of t-ROSCs during OHCA and its impact on outcome. Defibrillator-recorded OHCA events were analyzed via dedicated software. The number of t-ROSC episodes and their overall durations were recorded. The study endpoint was the good neurologic outcome at hospital discharge. Among 285 patients included in the study, 45 (15.8%) had one or more t-ROSCs. The likelihood of t-ROSC occurrence was higher in patients with a shockable rhythm (*p* = 0.009). The cumulative length of t-ROSC episodes was significantly higher for patients who achieved sustained ROSC (*p* < 0.001). The adjusted cumulative t-ROSC length was an independent predictor for good neurological outcome at hospital discharge (OR 1.588, 95% CI 1.017 to 2.481; *p* = 0.042). According to our findings and data from previous studies, t-ROSC episodes during OHCA should be considered as a favorable prognostic factor, encouraging continuing resuscitative efforts.

## 1. Introduction

Despite huge efforts in improving education and developing new technologies, out-of-hospital cardiac arrest (OHCA) continues to have a dismal prognosis [1]. In this dramatic and discouraging scenario, some conditions have shown to accurately predict a better survival probability: cardio-pulmonary resuscitation (CPR) provided by bystanders before emergency medical service (EMS) arrival, ventricular fibrillation or tachycardia as first detected cardiac rhythm, and the occurrence of return of spontaneous circulation (ROSC) in the prehospital setting [2]. Unfortunately, ROSC is often not sustained, such that after a variable period of restored spontaneous circulation, the patient may return into cardiac arrest. These temporary periods of return to spontaneous circulation (t-ROSC) that may occur during resuscitation from cardiac arrest (CA) have been previously found to be predictive of survival to hospital discharge [3]. Given the importance of these events, Utstein guidelines recommend to document the occurrence and duration of any ROSC event during CPR as a core data element [4]. 

For some years, technology integrated into defibrillators is allowing to document, in addition to the analysis of the electrical cardiac activity (ECG), the real-time modification in transthoracic impedance (TTI). TTI allows for assessment of the quality of CPR performance in the post-event period by the analysis of provided chest compressions and ventilations [5]. Moreover, the contemporary EGC and TTI analyses allow for identification of any t-ROSC periods occurring during the resuscitative maneuvers. Only a few studies analyzed t-ROSCs through the ECG and TTI traces during OHCA [6] and, to the best of our knowledge, the relationship between occurrence and duration of t-ROSCs and CA outcome has not been prospectively explored yet.

In the present investigation, we hypothesized that, in addition to already studied variables known to affect the CPR quality or the outcome of OHCA [7,8,9], the number and duration of t-ROSCs during OHCA would be related to short- and long-term CA outcome. For this purpose, we analyzed the ECG and TTI traces of 311 consecutive adult OHCA patients. 

## 2. Methods

### 2.1. Study Design and Population

This was a prospective observational study. All consecutive OHCA patients treated by the EMS teams of Trieste (Italy) from 1st January 2013 to 30th June 2015 were included. Patients were excluded if: (1) they were in pediatric age (<18 years); (2) a substantial lacking of data (e.g., failure in associating a record to a specific OHCA episode, ECG and TTI traces not accurately recorded due to delayed defibrillator pads connection) was found during the analysis of real-time recorded OHCA events; (3) recorded OHCA events lasted less than 5 min in the presence of asystole and ROSC was not obtained. 

Patients’ data collection was approved by the Institutional Ethical Committee within the Italian Registry of Cardiac Arrest-RIAC study [10]. All collected data were anonymized and handled only by the researchers and for the research purposes only. Data were stored on an electronic file, protected by a password. 

### 2.2. Characteristics of Study Setting and OHCA Management 

All EMS teams were trained according to European Resuscitation Council (ERC) guidelines [8] with particular emphasis on the importance of ensuring high quality CPR (i.e., appropriate rate, depth, and recoil in chest compression, effective ventilation, early defibrillation, and minimization of pauses to ensure the higher chest compression fraction). Ambulances were staffed with registered nurses trained in providing CPR, defibrillation, and intravenous adrenaline administration. In addition, emergency physicians moving by “rapid response cars” provided advanced intervention, such as mechanical CPR (LUCAS 2™, JOLIFE AB, Lund, Sweden), tracheal intubation or supraglottic devices positioning, and other drug administrations. Both nurse-staffed ambulances and rapid response medical cars were equipped with LP-12 or LP-15 defibrillators (Physio-Control, Redmond, WA, USA).

Upon arrival at the CA scene, the EMS team started or continued CPR (when it was already ongoing by bystanders or other healthcare providers). Defibrillator pads were promptly connected to check ECG rhythm and provide early defibrillation, when appropriate, thus activating continuous ECG and TTI recording. Mechanical chest compression and tracheal intubation were subsequently implemented, based on physician’s decision. 

Patients were transported to a single hub-hospital able to ensure post-resuscitation care including percutaneous coronary intervention (PCI) and targeted temperature management [11].

### 2.3. CA Data Processing 

After completion of the resuscitative maneuvers and patient management, the LP-recorded CPR events were anonymized through an automatically generated code and data were transferred by modem to a central server for subsequent analyses. 

**Figure 1:**
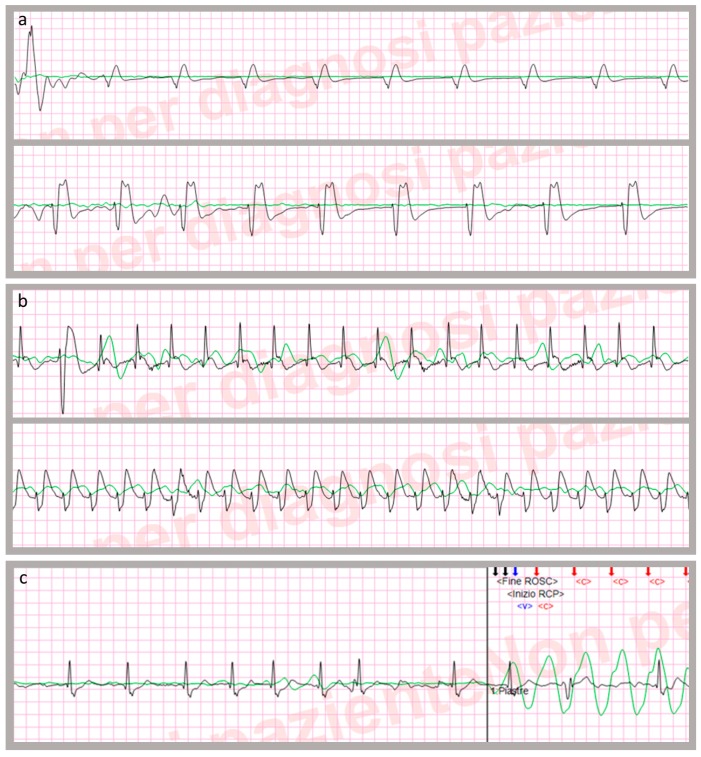
(**a**) Examples of pulseless electrical activity (PEA) in two different patients. (**b**) Examples of return of spontaneous circulation (ROSC) in two different patients. (**c**) Example of resumption of cardio-pulmonary resuscitation in a patient who had a transitory ROSC. Green line: transthoracic impedance signal. Black line: electrocardiographic trace. <C>: chest compression. <V>: ventilation.

Recorded data were analyzed via the Code-Stat^TM^ software (release 9.0, Physio-Control Inc., Redmond, WA), which displayed all relevant CPR events tagged in real-time by EMS teams (e.g., all ROSC episodes, intubation, defibrillation attempts, ECG and TTI traces) on continuous graphs reflecting the actual time of the events. In the present investigation, no manual modification of the Code-Stat reports was performed. To represent the TTI variations, a flat line was shown when no change affected the impedance, whereas a deflection of variable amplitude documented a temporary change in TTI related to lung inflation, left ventricular systole, and chest compressions [12,13,14]. Based on that, Code-Stat automatically recognized chest compressions and ventilations, whose curves were better displayed by activating a high-pass (1.5 Hz lower cut-off) or low-pass (0.5 Hz upper cut-off) filter, respectively [5,12,14,15]. Several variables were obtained to describe the main CPR quality parameters (Table 1) [12,16,17]. Moreover, the first ECG rhythm associated with OHCA, namely asystole, pulseless ventricular tachycardia (VT), ventricular fibrillation (VF), or pulseless electrical activity (PEA), was documented. PEA was defined as QRS-complexes without blood flow-induced changes in TTI [5,12] (Figure 1a). 

Studies have proved that cardiac contractions determine coincident modifications in TTI signal with high sensitivity and specificity [18,19,20], which permit differentiation of PEA from pulse-generating rhythms [20]. On this basis, all stages of CPR in which CodeStat displayed an interruption of chest compressions in the presence of an organized ECG activity were identified and analyzed. When QRS-associated modifications (“bumps”) in the TTI signal were found (Figure 1b) the condition was screened as a potential ROSC situation and classified as follows:

- “transitory” (t-ROSC), when the condition lasted more than one minute (irrespective of their duration or the moment they occurred during CPR) with resumption of chest compressions at a later time [6,21] (Figure 1c).
- “sustained” (s-ROSC), if the condition persisted until arrival at the emergency department [4].

As discussed above, conditions lasting less than one minute were not considered as ROSC at all. For each CPR episode, the number of t-ROSC episodes as well as their overall duration were recorded; duration and number were recorded as 0 when no t-ROSC occurred. 

### 2.4. Other Collected Variables

Data on patient demographics (e.g., age, sex) and prehospital variables (e.g., intubation, ROSC, administered drugs) were obtained from EMS documentations and, in case of any doubt, by interviewing the EMS teams. For example, a double check based on the EMS documentation and teams’ interview was adopted to verify the possible restoring of palpable pulse or spontaneous breathing during t-ROSC periods. 

An estimation of the time of OHCA onset was obtained by crossing the data of the dispatch center (recorded emergency calls, radio communications, and dispatch data) and EMS teams (medical records and interviews). The time between the OHCA onset and the start of CPR documented by LP defibrillators, whose internal clock was set up monthly based on the dispatch center official clock, was defined as the OHCA-CPR interval. 

The study endpoint was the good neurological outcome (Cerebral Performance Category [CPC] ≤ 2 [22]) at hospital discharge.

### 2.5. Data Analysis

Continuous variables were displayed as mean, standard deviation (SD), and median, and nominal variables as number and percentage. Unadjusted comparisons between groups were analyzed via χ^2^ test, unpaired Student’s *t*-test or Mann-Whitney’s U-test, as appropriate. 

Stepwise multiple logistic regression models were used to examine the independent association between cumulative t-ROSC length and the considered outcome, while controlling for variables resulting related to good neurologic outcome with a *p*-value < 0.1 in bivariate analyses. The polythomic variable related to the first documented ECG rhythm was recoded as dicothomic variable (0: nonshockable (asystole and PEA); 1: shockable (VF/VT)). During the assumption tests, no more than little or no correlation was shown between the considered variables (Pearson’s *r* < 0.3). In order to obtain a normal distribution for data showing excessive skewness or kurtosis, a square-root transformation was done on the OHCA-CPR interval, CPR time, ventilation rate, and t-ROSC length. The coefficient of determination of the regression model was calculated based on the Nagelkerke *R*^2^. The Hosmer-Lemeshow test for logistic regression was used to assess goodness-of-fit.

Statistical analysis was performed using IBM software SPSS Statistics, release 24.0 (Armonk, NY, US: IBM Corp.). For all tests, an alpha level of *p* ≤ 0.05 was set for statistical significance.

## 3. Results

During the study period, a total of 340 patients were rescued by EMS teams due to OHCA. Twenty-nine cases (8.5%) were excluded due to their insufficient quality of ECG and TTI recording. Among the 311 OHCA episodes with complete TTI and ECG data, two were excluded because were pediatric and 24 because they presented asystole CPR lasting less than 5 min without any ROSC. After applying the exclusion criteria, 285 OHCA patients constituted the final study population. Data on OHCA-related variables are described in Table 2. 

In 45 patients (15.8%), one or more t-ROSC events were documented. In all patients with Code-Stat documented t-ROSC episodes, the EMS medical records reported episodes of temporary palpable pulse restoration during CPR, while no patient restored spontaneous breathing during the t-ROSC periods. In patients with documented t-ROSCs, the CPR time was 24′:49″ ± 13′:34″, while the cumulative length of t-ROSC was 10′:35″ ± 9′:14″. The likelihood of t-ROSC occurrence was higher in patients with shockable rhythms. In patients in which t-ROSCs occurred, the ventilation rate was significantly higher and the uninterrupted chest compression rate significantly lower, whereas no difference was found for the other study variables (Table 3).

Overall, 81 (28.2%) patients achieved s-ROSC (age 72.5 ± 15.5 years). Among 45 patients who obtained one or more t-ROSCs, 29 (64.4%) achieved s-ROSC. The cumulative length of t-ROSC episodes was significantly higher for patient who achieved s-ROSC (declared dead: 0:32 ± 2:18; achieving s-ROSC: 4:34 ± 8:45; *p* < 0.001). 

Sixty patients died before hospital discharge and two were transferred to other hospitals without documented CPC score. Sixteen patients (5.7%) were discharge from the hospital with a good neurological outcome. Lower age, shorter CA-CPR interval, CPR started before EMS arrival, shorter CPR time, VF/VT as first documented rhythm, higher ventilation rate, and occurrence of t-ROSCs and their cumulative length were associated with good neurological outcome at hospital discharge (Table 4). 

Variables related to good CPC with a *p*-value < 0.1 in bivariate analyses (i.e., age, OHCA-CPR interval, CPR started before EMS, CPR time, shockable ECG rhythm, ventilation rate, and t-ROSC length) were inserted in the logistic regression model. The adjusted cumulative t-ROSC length was an independent predictor (OR 1.588, 95% CI 1.017 to 2.481; *p* = 0.042) for good CPC at hospital discharge (Table 5). Hosmer-Lemeshow test suggested that the model had a good fit (*χ*^2^ = 1.550; *p* = 0.992). 

## 4. Discussion

This study showed that the contemporary analysis of ECG and TTI curves allows to evaluate, in addition to other CPR quality variables, the occurrence and the duration of t-ROSC episodes in OHCA patients. The cumulative length of t-ROSC episodes was found to be significantly related to higher odds for sustained ROSC and a better neurological outcome was documented in patients who experienced t-ROSCs. To our knowledge, this is the first study that showed this relationship by identifying t-ROSCs with TTI and ECG analysis.

Some limitations of this study should be highlighted before discussing our results. First, the study had a retrospective design and enrolled a rather small sample in a single Emergency Medical Service. Second, some OHCA events presented substantial missing data because of technical difficulties in data storage or transmission after CPR monitoring. Although these problems occurred accidentally during the whole study period, we were forced to exclude 8.5% of OHCA episodes from the research. Third, an overall poor outcome was observed. Although we enrolled a particularly aged population, the presence of comorbidities may have affected the final outcome. In addition, postcardiac arrest syndrome is responsible for the majority of in-hospital deaths after CA [11], but data on the process of care following s-ROSC (e.g., ICU treatments) were not available in this study; consequently, the possible impact of post-resuscitative care on patient outcome was not taken into account. Fourth, the employed software did not allow quantification of the amplitude of impedance variations, such that for t-ROSC analysis, the value of TTI variation (“bump”) associated with QRS complexes was not measured as previously described [5]. Finally, several variables potentially affecting the OHCA outcome were not taken into account. For example, the EtCO_2_ was not systematically monitored in our population, such that this variable was not considered in data analysis. Similarly, complete data about the drugs delivered during CPR was available only for a minority of patients; therefore, these variables were not considered in the analyses. All the above limitations limit the internal validity of the study and may limit the generalizability of the study findings to a more universal population (external validity). 

Previously, similar t-ROSC incidence was reported and it was suggested that the occurrence of t-ROSC should be always considered as a strong warning for a favorable outcome potential [3,6]. A key point to consider is that the occurrence of any restoration of spontaneous circulation during CPR, even if short or unstable, is able to ensure a markedly higher tissue perfusion pressure compared to chest compressions [23]. According to Skogvoll et al. [6], t-ROSC represents—together with asystole, VF/VT, and PEA—one of the dynamic and transient states during resuscitation of OHCA patients; these states may often change over CA time, either spontaneously or as a result of resuscitative efforts. The occurrence of some changes is more likely than others (e.g., VF can evolve to asystole or s-ROSC depending on time to first shock and quality of CPR) and significantly affects survival [24]. T-ROSC is probably the most desirable transient state during resuscitation, being related to the presence of a palpable pulse and thus permitting the interruption of the low-flow state related to chest compression. The restored spontaneous circulation is the premise for a better brain and heart tissue perfusion. In the present study, we noticed that two-thirds of patients who regained ROSC at some point during CPR achieved sustained ROSC and that the occurrence of t-ROSC was an independent favorable prognostic factor for a good neurological outcome. 

We noticed a significantly higher prevalence of t-ROSC in patients with VF/VT compared to patients with asystole or PEA as a first detected ECG rhythm. However, the possible change of these rhythms over time, as well as the pulseless ECG rhythms that led to resumption of resuscitation at the end of t-ROSC, were not analyzed.

Several factors related to the underlying cause of CA or to provided interventions (e.g., administration of adrenaline) [25] may enhanced undesirable state transitions from t-ROSC to a new cardiac arrest. Any effort should therefore be acted to maintain the ROSC condition once it has occurred, so that, ideally, each t-ROSC possibly becomes an s-ROSC. Strategies should consider prompt support of the expected myocardial dysfunction (pursuing optimal arterial pressure target by considering the administration of inotropes and vasopressors), effective support of breathing avoiding the detrimental effects of both hypoxia and hyper- or hypoventilation [26], and identification and treatment of potentially reversible causes of cardiac arrest that may continue to be present despite the ROSC. For example, even brief t-ROSC intervals could allow EMS teams to record a 12-lead ECG and, if a myocardial infarction is diagnosed, to make the decision to provide thrombolysis [23]. Furthermore, since most advanced treatments and monitoring are not readily available in the prehospital setting, t-ROSC occurrence should be a critical factor to decide the patient’s transport to the hospital [3], allowing for further interventions, such as advanced cardiac output monitoring, PCI, or extracorporeal life support (ECLS) [27,28]. The availability of mechanical chest compression devices may extend this indication to patients with ongoing CPR after t-ROSC.

In OHCA patients, recognizing ROSC only through pulse checking may be challenging and time consuming due the possible low cardiac output, such that it is theoretically possible that rescuers prolong CPR interruptions beyond the recommended ten-second limit. The development of methods for automated determination of the presence or absence of cardiac contraction was prompted several years ago and TTI waveform analysis has shown a high discriminative power in recognizing pulse-generating rhythms in both animal and human studies [19,20]. Unfortunately, the feasibility of TTI analysis is currently not supported by medical devices for a real-time use during resuscitation. Industry should be solicited to develop methods for making this technology available. Meanwhile, given the importance of prompt recognition of any ROSC event during resuscitation, it is desirable for devices able to potentially increase their detection (e.g., end-tidal CO_2_ [8] or point-of-care echocardiography [29]) to be adopted. 

A final consideration on our results should be made acknowledging the lack of an independent impact of some CPR quality variables on patient’s outcome and, in particular, of the chest compression fraction, which is considered one of the most important CPR quality measures. Overall, we observed a good agreement with guidelines recommendation (e.g., chest compression fraction of 70%), thus we assume that this widespread good quality may have limited the impact of CCF as a predictor of outcome. Moreover, three quality parameters depending on the procedures (chest compression fraction, uninterrupted chest compression, and ventilation rate) were significantly different in t-ROSC patients; further studies are needed to detect their potential impact on outcome.

## 5. Conclusions

In the studied population, we found that the cumulative length of transitory ROSC events during CPR, identified by ECG and TTI analysis, was independently predictive of a favorable outcome. According to our findings and data of previous studies, any ROSC event should be promptly recognized during CPR. T-ROSC occurrence and duration can be interpreted as a favorable prognostic factor and should encourage the provision of high-quality CPR, even when multiple t-ROSC events alternate with new episodes of cardiac arrest. 

## Figures and Tables

**Table 1:** Definition of study variables related to CodeStat analysis.

Start of cardiac arrest episode	The first therapeutic event after EMS arrival and corresponding to the beginning of CPR manoeuvres (e.g., first recorded chest compression or first rhythm analysis)
End of cardiac arrest episode	The last registered chest compression, coinciding with having obtained a sustained ROSC or patient declared dead.
CPR time	Length of low-flow time (interval between “start” and “end” of CPR episode, after subtracting cumulative t-ROSCs duration)
Uninterrupted chest compression rate	The mean chest compression frequency per minute provided during uninterrupted chest compressions cycles, considering consecutive compressions which follow one another with pauses of less than 1.5 s
Chest compression fraction	The ratio between time spent delivering uninterrupted chest compressions and CPR time
Ventilation rate	The mean ventilation frequency per minute calculated by dividing the total number of ventilations by CPR time

EMS: emergency medical service; CPR: cardio-pulmonary resuscitation; t-ROSC: transitory return of spontaneous circulation.

**Table 2:** Main characteristics of the study population.

Variable	Data
Age (years) ^§^	74.1 ± 14.7 (76.0)
Sex (male) ^¥**^	178 (64.5%)
OHCA–CPR interval (min:sec) ^§^	9:12 ± 6:00 (8:00)
CPR started before the EMS arrival ^¥*^	44 (15.5%)
CPR time (min:sec) ^§^	23:40 ± 14:00 (21:50)
First documented ECG rhythm ^¥^	
VF/VT	81 (28.4%)
asystole	111 (38.9%)
PEA	93 (32.6%)
Chest compression fraction (%) ^§^	70.0 ± 12.4 (72.4)
Uninterrupted CC rate (CCs min^−^^1^) ^§^	115.4 ± 18.4 (110.1)
Ventilation rate (ventilations min^−^^1^) ^§^	7.7 ± 3.9 (7.1)
t-ROSC: number of events ^¥^	
0	240 (84.2%)
1	30 (10.5%)
2	7 (2.5%)
3–5	8 (2.8%)
t-ROSC: cumulative length of events ^§***^	10:35 ± 9:14 (7:32)
Sustained ROSC ^¥^	81 (28.2%)
Cumulative outcome at hospital discharge ^¥****^	
CPC 1	15 (5.3%)
CPC 2	1 (0.4%)
CPC 4	3 (1.1%)
CPC 5 or dead	267 (94.3%)

OHCA: out-of-hospital cardiac arrest; CPR: cardio-pulmonary resuscitation; EMS: emergency medical service; ECG: electrocardiographic; VT: ventricular tachycardia; VF: ventricular fibrillation; PEA: pulseless electrical activity; CC: chest compression; t-ROSC: transitory return of spontaneous circulation; CPC: Cerebral Performance Category. §: mean ± standard deviation (median); ¥: number; percentage. *: *n* = 284. **: *n* = 276. ***: *n* = 45. ****: *n* = 283.

**Table 3:** Relationships of CA-related variables with t-ROSC occurrence.

Variable	No t-ROSC Event (*n* = 240)	One or More t-ROSC Events (*n* = 45)	*p*-Value
Age (years) ^§*^	74.4 ± 15.0 (76.5)	72.9 ± 12.7 (72.0)	0.547
Sex (male) ^¥*^	150 (64.9%)	28 (62.2%)	0.728
OHCA–CPR interval (min) ^§^^**^	9.2 ± 5.7 (9.0)	9.0 ± 7.7 (7.0)	0.858
CPR started before EMS ^¥**^	36 (15.1%)	8 (17.8%)	0.644
CPR time (min:sec) ^§^	23:24 ± 14:06 (21:30)	24:48 ± 13:30 (23:00)	0.549
VF/VT as first documented ECG rhythm ^¥^	61 (25.4%)	20 (44.4%)	0.009
CC fraction (%) ^§^	70.4 ± 11.9 (72.4)	67.6 ± 15.0 (73.4)	0.250
Uninterrupted CC rate (CC/min^−1^) ^§^	116.4 ± 18.5 (111.3)	110.1 ± 17.2 (106.5)	0.035
Ventilation rate (breaths/min^−1^) ^§^	7.1 ± 3.5 (6.7)	10.4 ± 4.7 (9.7)	<0.001

OHCA: out-of-hospital cardiac arrest; CPR: cardio-pulmonary resuscitation; EMS: emergency medical service; ECG: electrocardiography; VT: ventricular tachycardia; VF: ventricular fibrillation; CC: chest compression; t-ROSC: transitory return of spontaneous circulation. §: mean ± standard deviation (median); ¥: n; %; *: *n* = 275; **: *n* = 283; ***: *n* = 284.

**Table 4:** Relationships of CA-related variables with outcome at hospital discharge.

Variable	Dead or CPC 3–5 (*n* = 267)	CPC 1–2 (*n* = 16)	*p*-Value
Age (years) ^§*^	74.7 ± 14.3 (76.0)	64.4 ± 18.6 (64.5)	0.006
Sex (male) ^¥*^	168 (65.1%)	10 (62.5%)	0.831
OHCA–CPR interval (min) ^§^^**^	9.3 ± 6.1 (9.0)	6.0 ± 4.0 (5.0)	0.032
CPR started before EMS ^¥**^	35 (13.2%)	8 (50%)	<0.001
CPR time (min:sec) ^§^	18:09 ± 11:47 (16:23)	11:13 ± 9:47 (8:26)	0.022
VF/VT as first documented ECG rhythm ^¥^	69 (25.8%)	12 (75.0%)	<0.001
CC fraction (%) ^§^	70.1 ± 12.3 (72.5)	69.3 ± 13.7 (73.4)	0.811
Uninterrupted CC rate (CC/min^−1^) ^§^	115.0 ± 18.2 (110.0)	121.2 ± 22.0 (114.7)	0.189
Ventilation rate (breaths/min^−1^) ^§^	7.2 ± 3.4 (6.9)	11.6 ± 5.7 (11.5)	0.008
t-ROSC periods (min:sec) ^§^	1:30 ± 5:12 (0:00)	4:43 ± 7:13 (2:22)	0.020
t-ROSC occurrence (yes) ^¥^	35 (13.1%)	9 (56.3%)	<0.001

CPC: Cerebral Performance Category; OHCA: out-of-hospital cardiac arrest; CPR: cardio-pulmonary resuscitation; EMS: emergency medical service; ECG: electrocardiographic; VT: ventricular tachycardia; VF: ventricular fibrillation; PEA: pulseless electrical activity; CC: chest compression; t-ROSC: transitory return of spontaneous circulation. §: mean ± standard deviation (median); ¥: n; %; *: *n* = 275; **: *n* = 283; ***: *n* = 284.

**Table 5:** Stepwise multiple logistic regression of good cerebral performance category (CPC) on study variables (likelihood ratio: 58.783; *R*^2^ = 0.572; *p* < 0.001).

Predictor	B (SE)	Wald Test	OR (95% CI)	*p*-Value
OHCA–CPR interval (min)	−0.908 (0.325)	7.808	0.403 (0.213 to 0.763)	0.005
CPR started before EMS (yes)	2.814 (0.812)	12.008	16.675 (3.395 to 91.902)	0.001
Shockable rhythm (yes)	2.397 (0.799)	9.004	10.987 (2.296 to 52.568)	0.003
CPR time (min)	−0.736 (0.245)	8.987	0.479 (0.296 to 0.775)	0.003
Ventilation rate (breaths/min^−1^)	1.204 (0.533)	5.097	3.334 (1.172 to 9.485)	0.024
t-ROSC length (min)	0.463 (0.228)	4.132	1.588 (1.017 to 2.481)	0.042
Intercept	−3.668 (2.039)	3.235	0.026 (/)	0.072

The variable “age” was excluded from the final model (*p* = 0.064). OR: odds ratio; CI: confidence interval; OHCA: out-of-hospital cardiac arrest. CPR: cardio-pulmonary resuscitation. EMS: emergency medical service. CA-CPR interval: time between OHCA and EMS-led CPR (minutes). Shockable rhythm: ventricular fibrillation or tachycardia as first documented ECG rhythm. CPR time: length of EMS-led treated cardiac arrest (minutes). t-ROSC: transitory ROSC.
